# Integrating foundation models with change detection to identify tasking for service robots

**DOI:** 10.3389/frobt.2026.1772005

**Published:** 2026-04-29

**Authors:** Eric Martinson, Igri Fishta, Devson Butani

**Affiliations:** College of Arts and Sciences, Lawrence Technological University, Southfield, MI, United States

**Keywords:** change detection, foundation models, Gaussian splatting, object detection, service robotics

## Abstract

Mobile manipulators show incredible promise as domestic service robots – interacting with a wide variety of objects using increasingly affordable hardware. But although perception, manipulation, and mobility have advanced, there remain fundamental challenges in making robots more useful. How can a robot proactively identify tasks that it can complete while supporting individual human preferences for how a home should be configured? We propose using foundation models to first detect what has changed and then select appropriate tasks for the service robot. Change affords action. Only those objects that have been interacted with need to be considered for tasking. Other objects, even if located in non-standard positions in the house, can be ignored. Open-vocabulary based object detection and neural radiance field models are used to identify changes corresponding to fixed phrases. Large language models then validate which tasks should be completed by the robot. Experiments are conducted on data collected by both mobile phone and Stretch 2 Mobile Manipulator, demonstrating general applicability to a wide range of applications in the home.

## 1 Introduction

The floorcare robots of today are only the first generation of domestic robots. Even so, they support busy families, improve general quality of life, and help elderly people stay in their own homes. Unfortunately, houses are unstructured environments with too many variables – many of which are unimportant for most tasks – so robotics companies have struggled to generalize other applications to homes, even within the narrower domain of elder care. Change detection can reduce this complexity by focusing attention on only those parts of the environment important to available robotic tasks. Meaningful change affords action. Take the example of a mobile manipulator that can detect and put away different categories of objects. Current best approaches to tasking are to treat every object in a predefined area as an object to be put away (e.g., floor (Wu et al., 2023a), table (Reister, et al., 2022), etc.). Only picking up objects from a single area does not usually justify the cost of the robot, even in healthcare. And current methods do not generalize well to support all areas of the house. What categories of objects should be picked up when using object detection? What if a person does not want some objects to be picked up, despite being in an unusual spot? Change detection can be a vital part of such decision making. It is not the only driver people use for deciding to act, but when change is noticed, people make conscious choices about whether to do anything about it.

Change detection in general is the challenge of identifying what is different between two environments. A robot, or just a person with a mobile phone, captures images throughout an environment, collecting data for a baseline “normal” condition. Then, in subsequent runs, images are compared to the baseline to identify what has changed. Such repeated visual observations of an environment are common in big data: people capturing temporally separate video streams with phones; facility security combining fixed camera with human patrols; robots cleaning or monitoring a home. The challenge is effectively processing these highly repetitive data to extract useful results. Event-based methods like object detection struggle with a lack of application specific training data, while anomaly-based methods have high false positive rates requiring significant human review. Indoor spaces further complicate the matter as they are often co-occupied by people, so they change constantly and have highly individual detection requirements (e.g., papers lying on the table are often unimportant in homes, but could be critical in a military or industrial setting). What is needed are new ways for incorporating context into the search, discarding that which a human observer would otherwise ignore.

**FIGURE 1:**
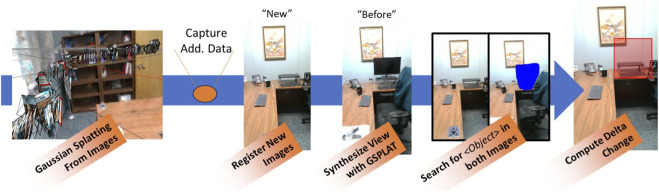
Steps of the process for estimating the delta change between different data captures in the same environment.

Addressing this challenge, we have developed a novel approach to change detection that identifies clean-up targets for a home service robot (Figure 1). Neural Radiance Field (NeRF) (Mildenhall, et al., 2021) or Gaussian Splatting (GSPLAT) (Kerbl, et al., 2023) enables the generation of baseline “normal” images from the same perspectives as newly collected images. These represent before and after images of the same location. They can be directly compared by applying detection/segmentation methods to both images to estimate delta change over time. A singular advantage of new foundation models like CLIP-Segmentation (Lüddecke and Ecker, 2022) and the Segment Anything Model v3 (Carion, et al., 2025) is their ability to search for generic objects, including phrases like “unexpected mess” or “small items”. Such flexibility means context can be incorporated into the search to find many object types with a single query. Conversely, using generic phrases can also capture many objects beyond those intended. By comparing open vocabulary outputs on both new and baseline images, the numbers of pixels, images, and object-level bounding boxes is reduced by more than 50%.

Fusing delta change outputs together into point clouds and applying clustering further improves performance and generates specific object pick-up targets. With data collected by a robot equipped with RGB-D and laser-based localization, change detection reduces the number of pickup targets by more than 50% while preserving the number of correctly detected changes. The same approach can also be deployed with just a handheld mobile phone without depth data, still reducing the number of pickup targets by more than 30%. Both data sources can be further analyzed with a large visual language model (LLM) to make the final decision about whether or not the object can be (or should be) picked up–thereby avoid interactions with objects that are too heavy or delicate to move.

This work builds upon previous efforts detecting change with Neural Radiance Field (NeRF) models (Martinson and Lauren, 2024) and fusing open-vocabulary based outputs to improve object detection (Martinson and Indurthi, 2025). Specific advances included here are: (1) integration of data fusion to improve change detection, (2) application of VLM’s to further refine outputs, (3) comprehensive evaluations of image- and cluster-level results on robot and mobile phone generated data.

## 2 Related work

### 2.1 Change detection – Classical methods

**FIGURE 2:**
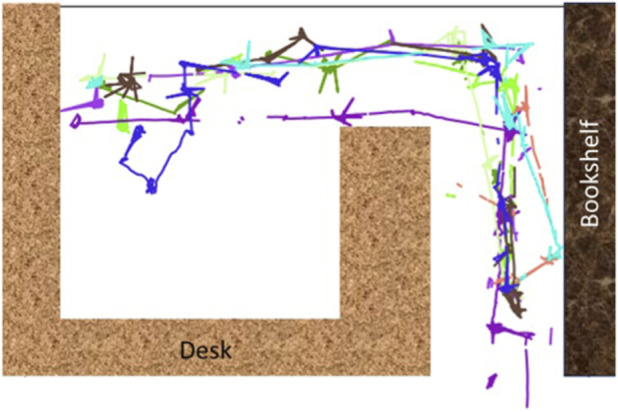
Colored lines represent the variations in reconstructed paths when using a robot to capture repeated data in the same environment.

Classically, change detection starts with image registration–once images are aligned, they can be compared through a wide range of methods (Lu et al., 2004). Static mounted security cameras are already registered, and so change has already been widely adopted by such systems (i.e., intruder detection). With moving cameras, the challenge is that cameras are never in exactly the same place twice (Figure 2 shows multiple paths by a robot through an indoor space while following the same waypoint trajectory). With sufficiently long distance, and high enough resolution (e.g., satellite and/or drone imagery), bundle adjustment is enough to align the images spatially and identify regions of overlap (Grodecki and Dial, 2003). But distance and downward angles, are crucial–impractical conditions for home monitoring.

With autonomous cars capturing images at street level, a difference of a few meters between captures is too large to overcome through digital alignment. Although bundle adjustment provides accurate relative locations to previous imagery, estimating change requires additional methods. Sakurada and Okatani (Sakurada and Okatani, 2015) use super-pixel-based features to build difference maps between the nearest image pairs to identify change. Alcantarilla et al. (Alcantarilla, et al., 2018) also focused on change between image pairs, but opted to reconstruct the pose from a dense reconstruction tailored specifically for outdoor environments. This method relies on relative distance to objects to blur some of the finer detail in the reconstructed image. Even then, there are large gaps, particularly at close range. Overcoming such noise in the reconstruction is then generally handled by a deep learning solution. With enough training data, deep learning enables a number of similar methodologies (Varghese, et al., 2018; Mandal and Vipparthi, 2022; Wanga, et al., 2023), some with no or only limited transformations on the “closest” spatially aligned image from baseline.

Most of the methods discussed so far represent “pixel-based” change detection, focusing on reconstructing an image mask of all change in each image. Without context, not having semantic descriptors of what has changed would make observations of what has changed in the home quite difficult. One possibility is to use an LLM to describe the regions identified in the mask (Ranasinghe, et al., 2024), but this does not guarantee a focus on only what has changed. More often, the solution is to train a separate neural network on subjects a human annotator marks interesting. With outdoor data, for example, change detection usually ignores seasonal or weather-related changes (e.g., trees that have lost leaves (Sakurada and Okatani, 2015), and snow-covered ground (Alcantarilla, et al., 2018)).

When the type of change being tracked can be defined by specific object categories (e.g., signs, cars, buildings, etc.), then object classification can help. Liu, Yang, and Lunga (Liu, et al., 2021) used a 4-class object classifier to describe the land below in satellite imagery, building a coarse 2D map of the ground from poorly registered images, and then comparing maps *post hoc* to find areas damaged by tornado activity. Goncz and Majdik (Göncz and Majdik, 2022) build semantic maps of both urban and indoor areas, comparing newly detected objects with the existing map. Wanga, Gaob, and Wang (Wanga, et al., 2023) skip building a map, but use semantic segmentation to extract object information that can be fed into a deep learning model. In all of these, however, object-based change is restricted to the narrow set of categories the original classification step could be trained on.

To improve upon the narrowness of object-based change detection, Obinata (Obinata, et al., 2023) proposed a novel, CLIP-based backend to grow the types of objects extracted. Unlike our work, Obinata requires training a full vision-language model to create application specific captions for images. Then, a vectorized description of the room is created from the combined captions. Change is estimated by comparing normalized vectors between runs. This approach is highly novel in its use of captioning, but still requires significant training and is subject to occlusions during the run (e.g., a count of detected computers drops because a chair is blocking more images than previously).

Using multi-turn dialog to refine search and segmentation with a LLM is another way to get at information about the scene. LISA (Lai, et al., 2024) and LISA++ (Yang, et al., 2023) used a modular approach of iterative refinements to handle attribute- and- or abstract reasoning when segmenting objects. SegLLM (Wang, et al., 2024) incorporates a visual memory to leverage previously generated masks during multi-turn dialogs. Other solutions connect LLMs with transformers architectures (Zhang H. et al., 2022) and Panoptic Scene Graphs (Zhou, et al., 2023) to achieve similar enhanced segmentation in response to user interaction.

### 2.2 Open vocabulary object detection

Numerous object detection systems based on training with labeled data have been deployed in robotics. These include image-based systems like Yolo (Ultralytics, 2023) as well as point cloud-based systems like PointNet (Qi et al., 2019). Labeling data to add new objects, however, remains a significant and expensive challenge. Martinson and Alladkani show how error-prone existing labelling processes are, and proposed leveraging large existing user bases at iRobot to rapidly train new object detection systems (Martinson and Alladkani, 2024). Without access to significant data, however, development of new object detection systems for robots is difficult. This is where a new generation of open vocabulary detectors like CLIP (Radford, et al., 2021), ALIGN (Jia, et al., 2021), and LiT (Zhai, et al., 2022). become important. Trained on matching images with the right caption, these are import base networks leading to many other applications. Lüddecke and Ecker (Lüddecke and Ecker, 2022) demonstrated how CLIP could be used to produce segmentation images, and researchers at UC Berkely went a step further to add language-based segmentation to 3D reconstruction (Kerr, et al., 2023). With large datasets like LAION-5B (Schuhmann, et al., 2022) supporting further research into the field, groups like Microsoft demonstrated how multimedia data could be directly integrated into an LLM with Kosmos-2 (Peng, 2022) or even synthesize new imagery from seed examples with Dall-E3 (Peng, 2022). A number of other multimedia LLMs have been striving for similar goals (Wu et al., 2023b), and they are now applied to robotics (Kawaharazuka, et al., 2024).

Uploading images for cloud processing with multimedia LLMs, however, is not mandatory for open vocabulary object detection systems. CLIP can also teach a classical detection network as part of teacher/student framework (Gu, et al., 2022). Or lightweight heads could be directly attached to image-encoder outputs, training the new heads against standard detection datasets to place bounding boxes around open vocabulary queries (Minderer, et al., 2022). Zang et al. (Zang, et al., 2025) take this a step further by integrating with an LLM, but instead of transmitting an entire image to the LLM, image features from ResNet are supplied to the LLM along with text context to localize important subjects related to the input context. These lighter weight models will also be used in this work–helping to improve precision after change detection was applied in Section 3.2.

## 3 Algorithms

**FIGURE 3:**
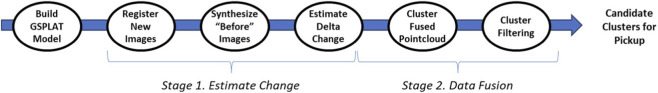
The entire change detection process can be broken into two stages: (1) estimating delta change per image, and (2) data fusion.

This work is divided into two stages (Figure 3). In the first stage, the focus is on detecting change at the image level using Radiance Fields or Gaussian Splatting to reconstruct images from the same vantage point at different times, and then using Open-Vocabulary Object Detection to compare the results. The second stage is then about fusing these data together into a point-cloud and processing the resulting clusters with an LLM to identify candidates for pickup with a mobile manipulator service robot.

### 3.1 Step 1 – creating a delta change image

Our approach to detecting change requires generating images from the same viewpoint at different times (Figure 1). This means two separate data collections. In the first instance, images captured along a path using either a handheld mobile phone or a mobile robot are registered into a common transformation space using Colmap (Schoenberger, 2026), and then used to build an image reconstruction model with either NeRF or GPLAT (Kerbl, et al., 2023). This work uses NerfStudio framework (Tancik, et al., 2023). By default, the Splatfacto implementation of GSPLAT is used for mobile phone data lacking depth images. Where depth data are available, such as when using a mobile robot, the depth-nerfacto method provides a better reconstruction. Either model enables dense image reconstructions of a baseline from an arbitrary viewpoint. Similar to previous image reconstruction-based approaches [8], GSPLAT and NeRF models are particularly good at reconstructing cluttered indoor spaces. Images captured at a later date can then be registered into the same transformation space as the baseline, and the NeRF model used to reconstruct the viewpoint of the newer image. The results are “before” and “after” images with which to estimate change.

**FIGURE 4:**
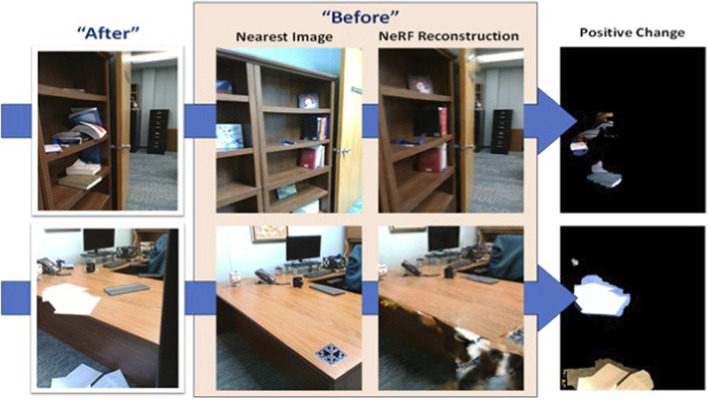
The “after” image is captured during a second run through the environment. The “before” image is the NeRF reconstruction of the same viewpoint. The “Nearest Image” is presented to show the closest image captured during the original baseline run. The final, “positive change” image is change detected using the query, “unexpected mess”.

The primary advantage to this approach is in being able to apply image segmentation methods to the same viewpoints at different times–then change is just the difference image between them. By contrast, Figure 4 compares a NeRF-based image reconstruction to the nearest image collected by angle. Whereas nearest neighbor images would require a hand labeled dataset to train a neural network to process, these before and after images can be deployed with any open-vocabulary based image segmentation network and arbitrary search strings.

These methods have been deployed with both hand-held mobile phone camera (iPhone 13 and 16 without depth) and a mobile robot equipped with laser-based localization and pan-tilt camera. The algorithm for creating these difference images is the same for both platforms: (1) build a Gaussian Splatting or NeRF model of the baseline, unchanged condition; (2) after capturing images at a later time, register new images with respect to the baseline using Colmap; (3) reconstruct images from the earlier baseline condition using the new image positions; (4) estimate change by combining a target search query with the open vocabulary segmentation model applied to both the generated image and the original; (5) subtract the baseline segmentation from the new segmentation and cluster the results.

#### 3.1.1 Capturing data for image reconstruction

**FIGURE 5:**
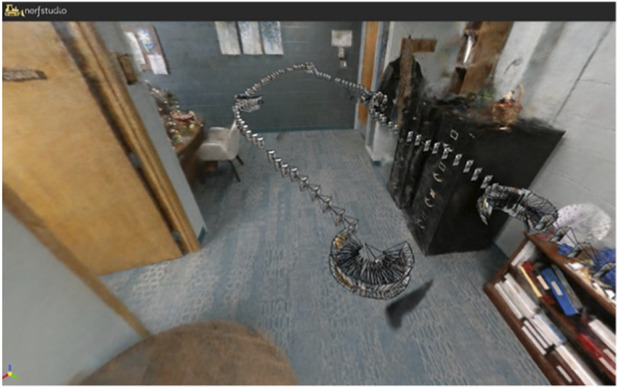
The path of images captured to build a baseline model of the office. The robot drove in straight lines, then stopped and sampled in many directions, which show up as clusters in this representation.

To create a baseline model, images are captured as a stream along a predetermined path (Figure 5). This work identified specific waypoints in each room that a person or robot would move to and then capture images with different pan and tilt values. Motion blur is a challenge. With the mobile robot, images were captured at each of 36 pan/tilt values per waypoint, moving the pan/tilt device completely before capturing the next image. More limited data were captured between waypoints at slow speeds to limit the number of images that might contain motion blur. With the phone camera, the operator simply made effort to move slowly. Note that data capture between waypoints was critical to image registration even if it introduced some blur.

In subsequent captures where change is being investigated, the robot or human operator move along a similar path but stop less often–capturing fewer images. It is possible to repeat the same stops, but this leads to significant computational overhead when registering large numbers images without clear advantage in detecting change. Note that repeating the exact trajectory is also not important, as can be demonstrated by traversing the route in reverse and sampling at different locations. However, the farther away from the original path, the more error in the resulting reconstruction later on.

#### 3.1.2 Generate before and after images

Given one or more new images to analyze, the first step is to localize these images of a potentially altered environment into the coordinate system of the original, baseline run. Initial attempts at image registration used ROS utilities for Adaptive Monte Carlo Localization (AMCL) (Gerkey, 2020) to localize the robot in a map from laser scan returns. Unfortunately, the resulting pose estimates have too high an error for an accurate image reconstruction. For better accuracy, the position of newly captured images from a second patrol through an environment are determined using another built-in Colmap utility. The process, described in the Colmap FAQ (Schoenberger, 2026), is to extract features from the new images, apply a vocabulary tree matching algorithm to identify matches between new and old images, then register them into the model. We apply the 1M vocabulary model created from the Flikr 100K dataset during tree matching to maximize the number of successfully registered images. AMCL estimates generated with the laser are then used to scale the Colmap returns using the built in geo-registration utility.

**FIGURE 6:**
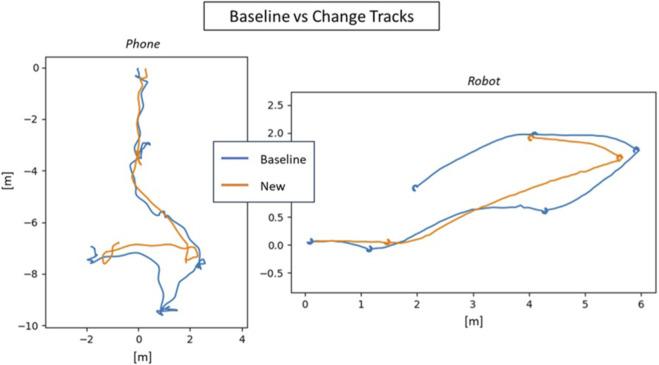
Top-down view of baseline track and new data capture track in two environments to demonstrate the repeatability of the image registration process.

Mobile phones do not have laser-based localization, but do have IMU measurements to provide pose estimates. This work leverages the Spectacular Rec app to record camera and IMU data, which were then processed with the Spectacular AI SDK (Spectacular AI, 2025) to extract images and camera pose estimates. The Spectacular AI SDK is loosely based on Seiskari et al. (Seiskari, et al., 2022) and is available on both IOS and Android devices. A key-frame distance of 5 cm was used during extraction. To register new images with old images, COLMAP was still used to align images between runs, and then image poses geo-registered with the Spectacular AI SDK generated pose estimates. Figure 6 shows relative overlap between runs for both the phone and the robot after completing this alignment process.

This approach is robust to modest amounts of environmental change. Objects were added and removed, windows opened, lighting changed, books and papers scattered, etc., and still most images were registered correctly. Erroneously localized images generally fell into two categories: either registration fails entirely or Colmap would find a highly erroneous position. Failures generally happened when the changed region accounted for the majority of the image (e.g., looking out a recently opened window), the image was blurry (e.g., from motion), or if there were very limited visual features to be recovered (e.g., a picture of a blank wall). Although early implementations removed “large jumps” in pose (Martinson and Lauren, 2024), the implementation presented here does not use a pose filter, instead depending on *post hoc* data fusion to remove error.

**FIGURE 7:**
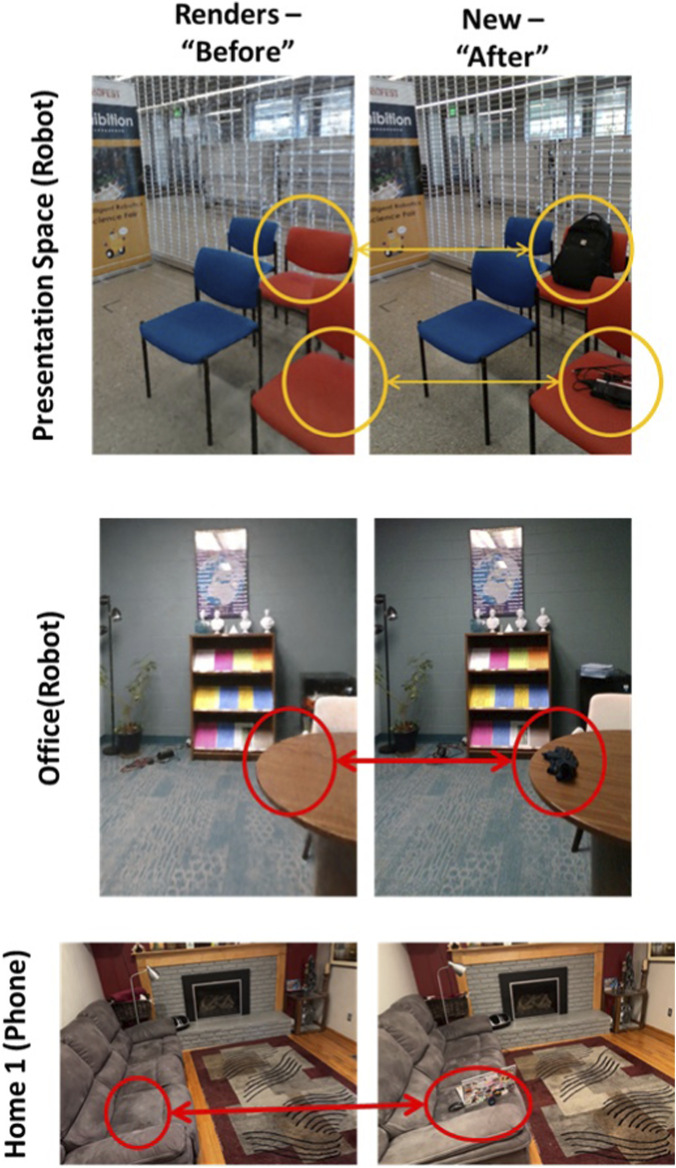
“Before” and “After” images highlighting the quality of the image registration and the image rendering in 3 environments.

The new images registered with Colmap are the “after” images to investigate for change. Given the specific pose and camera parameters identified for each image, Nerfstudio then generates a “before” image representing the environment during the baseline run. Figure 7 highlights before and after images created in several environments.

#### 3.1.3 Applying object segmentation

**FIGURE 8:**
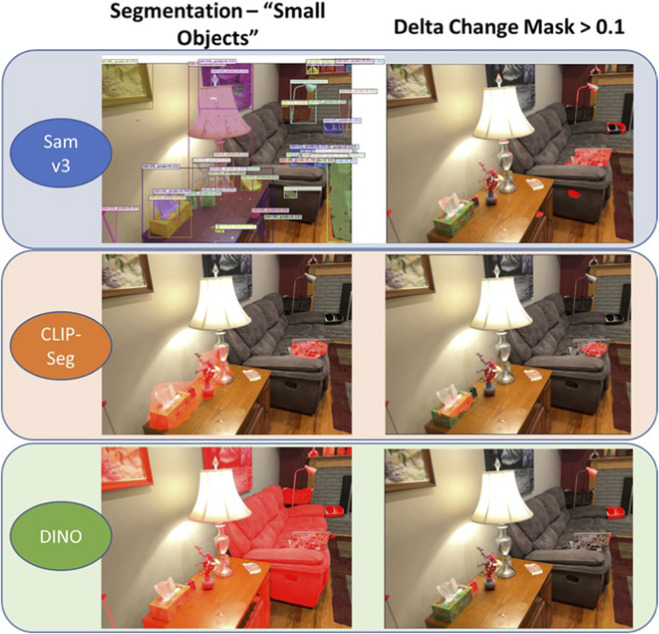
Comparison of three different algorithms evaluated for use with creating delta change masks. CLIP-Seg and SAM-v3 work reasonably well with generic queries like “small items”, but DINO does not differentiate well between the items that exceed threshold.

The next step is to perform image segmentation on both the before and after images using open-vocabulary based segmentation. This work was evaluated with both CLIP-Seg and SAM v3. Unlike traditional models that require manually annotated datasets for training on particular tasks, these foundation models are trained on a very wide range of images and associated textual descriptions, allowing them to generalize across a range of visual concepts - learning representations of images and texts in a shared embedding space so that images can be matched with relevant text and *vice versa*. Of particular interest for change detection is the ability to localize objects associated with generalized, nonspecific text like “trash”, “mess”, “small items”, “clutter” – items that people, and by extension the robot, should interact with without knowing exactly what they are. Initial testing was also conducted with Yolo-World and DINO, each coupled with a SAM v2 model to generate segmentation images. Although these methods are popular in robotics, excelling at detecting narrow object queries (e.g., backpack, cup, book, etc.), neither method worked well when localizing objects with nonspecific prompts. Take “small items” as an example. Where CLIP-Seg and SAM v3 might have some large objects exceed threshold, smaller items like laptops and Kleenex exceed that threshold by significant margins. DINO, however, has a lot of objects ranked with similar scores so the resulting change mask does not differentiate between new and old objects well (Figure 8).

Once both the before and after images have been processed with object detection/segmentation, the next step is to generate a delta change image. This requires identifying the direction of change (Wanga, et al., 2023). Is the operator searching for missing objects? Or something that has been added to the scene? In our experience, each query is associated with a single direction. For instance, when searching for known objects, you might search for TVs/monitors/computers that are missing. Adding a new object of that type is unimportant when determining the need for reporting change. Conversely, if searching for something unexpected, the goal is to find things that have been added to the environment. The indicated directionality controls the direction of a Delta function:

$$delta_{change}=\left\{\begin{array}{cc} clip_{before}-clip_{after} & if\;missing\\ clip_{after}-clip_{before} & if\;added \end{array}\right.$$

While in theory an absolute value function could be applied to detect any direction of change, absolute value operator results were found to be too noisy. this may be due to how models are trained on image captions, which only focus on the most interesting part of the scene rather than finding all instances in each image.

#### 3.1.4 Bounding box generation

**FIGURE 9:**
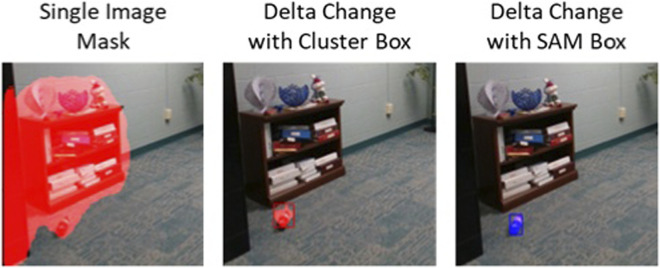
Starting with single image mask for the “small items” query (Left). A delta change image is created for this image, clustered, and a bounding box drawn around it (Middle). The same coordinates can also be used as a seed with SAM for a better fit (Right).

Delta change images do not contain bounding boxes even if the original methods (e.g., SAM v3) generate bounding boxes. Bounding boxes, however, will be useful later for identifying which images contributed to extracted objects. To generate bounding boxes, Density-Based Clustering (DBSCAN) (Ester, et al., 1996) is used to generate clusters from pixel row/col coordinates. Clusters with less than 50 pixels are discarded. Bounding boxes per cluster are then calculated from pixel membership (Cluster Box). For tighter fits around the object, SAM can also be used with the DBSCAN box as a seed (Figure 9).

### 3.2 Step 2 - Data fusion

Step 1 focused on reducing the number of objects per image to be considered. It used foundation models in the form of open-vocabulary object detection methods. Per image, however, it is difficult to distinguish between real change and errors with “before” image synthesis. Step 2, therefore, focuses on Data Fusion combining evidence from different observation angles to improve classification likelihoods. This work follows a similar methodology to that deployed in (Martinson and Indurthi, 2025), generating point clouds, building clusters, and then applying filters to remove false positives.

#### 3.2.1 Clustering

The delta change image is applied as a mask to the associated Depth image, generating a cloud of points per image with delta change > threshold (i.e. 0.1). All points preserve location {x,y,z} in a global coordinate frame and the likelihood of change. Points from all delta change images exceeding threshold are fused together into a single point cloud per input text string per run. Tracking two different prompts (i.e., “trash” and “small items”) will generate two different point clouds.

The combined point cloud includes points on both “changed” objects and false positive, or unchanged objects, detected during the second scan. DBSCAN is applied to extract object hypotheses from the cloud. The full point cloud, however, has too many points to run clustering, so 1-cm Voxel-based down sampling is first run on the full point cloud to reduce the number of points and associated load. Probabilities are summed together during the down sampling step and used as sample weights when clustering. Down sampling has an added benefit in that the number of points remaining on the object should now be related to the visible surface area of the object rather than number of images captured. Although not used here, this can provide significant value for filtering objects with estimable sizes (Martinson and Indurthi, 2025). DBSCAN parameters for the reduced cloud are EPS equal to 5x the voxel size (experimentally determined) and a neighborhood size of 500.

**FIGURE 10:**
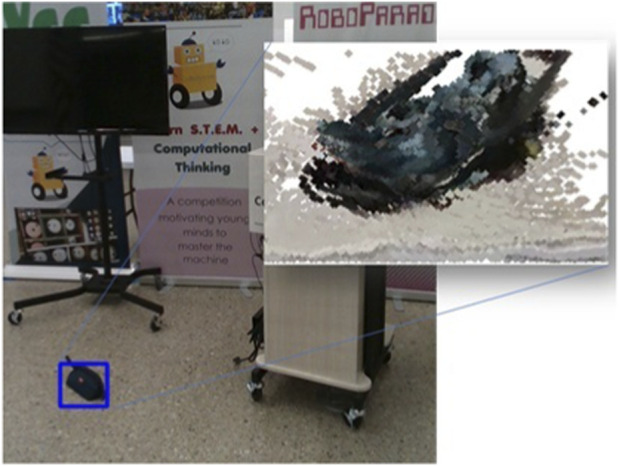
Extracted point cloud for an object that was identified with change detection and resulted in a distinct cluster.

Figure 10 shows an example point cloud cluster visualized in 2D.

#### 3.2.2 Merging clusters

A delta change mask is estimated on color images and then applied to depth images. What if the depth image is noisy (e.g., using synthetic data with mobile phone images)? Or the surface is untextured (e.g., a table or wall)? This can lead to multiple clusters being created from the same set of images and extracted bounding boxes–but located spatially at different depths. To rectify this situation, clusters are compared to the original extracted bounding boxes.

Let S_A_ and S_B_ be the set of detected change bounding boxes contributing to cluster A and B respectively. If the intersection of S_A_ and S_B_ is >50% of the length of both boxes, then the two clusters are merged together. With the synthetic depth data, this approach reduces the number of clusters by ∼20%.

#### 3.2.3 Filtering remaining clusters

After merging clusters, remaining clusters are scored with several different functions to provide a means of removing additional false positives. These functions include both statistical methods and LLM image analysis:

- Mean Probability: The mean probability of all points contributing to cluster A
- Percent Valid: Let *I*_*A*_ be the set of images that, based on camera parameters (focal length, center pixel, and height/width) and image pose, should be able to view centroid of cluster A. Let *V*_*A*_ be the subset of *I*_*A*_ that contains a bounding box contributing to cluster A. Then the percentage of valid change images is:

$Pct_{valid}=\frac{\left|V_{A}\right|}{\left|I_{A}\right|}$

- LLM Pickup: *I*_*A*_ is split randomly into sets of three non-overlapping images, with identified bounding boxes drawn around the area of interest in each. These images are sent to a large language model (Llama Scout 70B (Meta, 2025)) running on a local GPU server along with the following query:

We are deciding on tasks for a robot that can pick small stuff up and put them away. The robot should pick up things left behind by people that used the room. This includes all small stuff that does not normally belong in this kind of room. The robot should not pickup decorations or expensive electronics. The object surrounded by the blue box in the provided image(s) has been identified by the robot as a candidate for picking up. Is the object surrounded by the blue box in these images an object that the robot should pick up and put away? Return an answer in JSON format as {‘is_pickup’: <True/False>}

**FIGURE 11:**
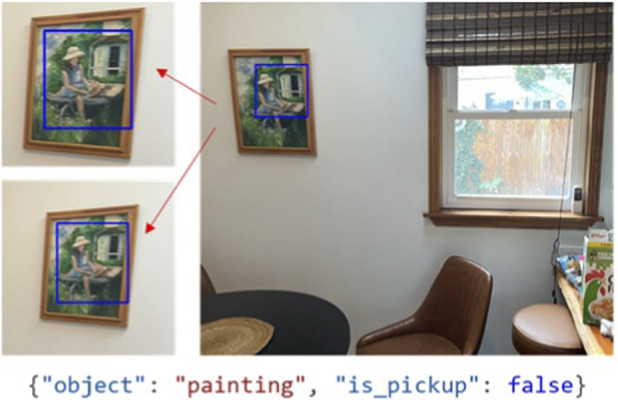
Example query to a LLM to request a decision on whether or not the object should be picked up. In this example, the painting is correctly recognized and the decision is not to pick up the object.

The language model returns a Boolean answer for every triplet. The pickup score is the percentage of answers marked as True. This filter is particularly important for deciding upon robotic action, as many items that are otherwise more challenging to reconstruct accurately like photos and painting (Figure 11) can be marked as off limits for a robot.

- Combo Percent Valid/LLM Pickup–Calculate the scores for each of the Percent Valid and LLM Pickup filters separately. Then multiply these likelihoods together to create a single value.

Each of the above methods are ways of scoring the resulting clusters. They generate values between [0,1] where a threshold can be applied to reach precision/recall/specificity targets. Other combinations of Mean Probability/Percent Valid/LLM Pickup were also tested, but were left out of the results as they offered no significant difference from their individual strongest component.

Note that we have also experimented with using the LLM to support change detection directly (i.e., are these two images different, or different in the region of interest). Initial investigations suggest that the models like Llama:Scout and Google:Gemma do not process rendered images well, leading to significant error when providing our before and after image pairs. We are actively investigating using the most similar image from the baseline run instead of a rendered image, but at present, asking the LLM about the suitability of the object for being picked up is by the far the most effective query.

## 4 Results

### 4.1 Evaluation datasets

Four datasets were collected: 2 with the mobile phone, and 2 with the Stretch-2 robot. Several datasets are depicted in Figure 7. Home spaces were collected with permission of the owner without people present.

- Home 1 (Mobile Phone) tracks changes in a living room space of approximately 5 m × 4 m. 407 baseline images were collected. 4 change tracks with ∼100 images each were collected across 2 days of recording. Tracked changes include dishes, toys, computers, cables, books, clothing and blankets.
- Home 2 (Mobile Phone) tracks changes across a dining/living room space of approximately 9 m × 3 m. 642 baseline images were collected. 4 change tracks with ∼150 images each were collected across 2 days of recording. Tracked changes include dishes, bags, and clothing.
- Presentation Space (Robot) is a simulated lecture space (∼4 m × 4 m) in a visually cluttered environment. Posters along all walls with pictures generate significant false positive detections with all classifiers. To account for people moving furniture and/or walls in this shared space, baseline data were collected at the beginning of each of 3 days of trials. 10 different change runs were completed, tracking phones, chargers, notepads, bags, soda bottles, outerwear (hat, gloves, jacket) – items commonly left behind in lecture halls. Baseline data have ∼350 images per run, while change runs have ∼180 images.
- Office (Robot) is a working office space of 6.5 m × 2.5 m characterized by a moderate level of visual clutter and frequent human activity. The space contains desks, shelving, personal items and office equipment that remain largely consistent over time, with variability primarily introduced by personal belongings of individuals using the office. Data were collected over multiple consecutive days to capture natural variations in object placement, while maintaining a largely stable scene structure. Change runs reflect realistic usage of the space, where items such as papers, chargers, soda bottles, gloves, backpacks and lunch boxes were temporarily left behind by occupants entering or working in the office. Because the office was still being used during data collection, some sampling images include people within the scene, requiring the system to tolerate partial occlusions and dynamic elements. Baseline data have ∼420 images per run, while changes runs have ∼200 images.

### 4.2 Algorithmic variations evaluated

6 different algorithms were tested in subsequent analyses, varying the choice of open-vocabulary object detection/segmentation methods and the use of a blur filter. Algorithms tested include CLIP-Seg and SAM3. Blur filtering was accomplished by calculating 2 different blur metrics (Tenengrad/Sobel, and FFT energy) and adding them together. The 30% of the images with the blurriest GSPLAT reconstructions are then removed before applying change detection.

The following algorithms all use a uniform 0.1 detection threshold to maximize the number of objects that could be detected. Multiple thresholds were tested initially, but this low threshold seemed to perform the best for both algorithms. SAM v3 would drop most generic prompts like “small items” with higher thresholds, whereas CLIP-Seg was less sensitive to the specific value.

- CLIP-Seg (Open Vocab) – CLIP-Segmentation without change detection or blur filtering to identify pixels with threshold >0.1 association to any provided prompt. Bounding boxes around regions of significant difference are estimated by first clustering pixels with DBSCAN and then using SAM v2 to expand to the visual edges of the cluster in the image.
- CLIP-Seg (Change) – CLIP-Segmentation with change detection, but no blur filtering. As above, it uses a 0.1 detection threshold, and DBSCAN + Sam V2 to estimate bounding boxes around detected objects
- CLIP-Seg (Change/Blur) – CLIP-Segmentation with change detection and blur filtering. As above, it uses a 0.1 detection threshold, and DBSCAN + Sam V2 to estimate bounding boxes around detected objects. Only images that pass the blur filter are included in the results.
- SAM3 (Open Vocab) – SAM v3 is used to identify all pixels and bounding boxes without change detection or blur filtering.
- SAM3 (Change) – SAM v3 is used to identify all pixels and bounding boxes with change detection active, but no blur filtering.
- SAM3 (Change/Blur) – SAM v3 is used to identify all pixels and bounding boxes with change detection active. The blur filter is applied before change detection to remove 30% of the blurriest reconstructed images.

### 4.3 Evaluation of change-based filtering

Change detection is essentially a filter. It removes unnecessary data from the processing pipeline. As demonstrated in Figures 4, 8, 9, there are many individual examples where change detection eliminates unchanged objects or surfaces from consideration. In principle, if two images were captured from the exact same position, but objects had been moved around, then the approach will filter out unchanged parts of an image. There are three sources of noise, however, that could cause significant problems.

1. Image Registration Error: If images are registered incorrectly, then the reconstruction will be different from the original. This happens most frequently with images that lack adequate texture for registration (e.g., walls). Coincidentally, poorly registered images lacking texture are also less likely to contain interesting change. COLMAP filters many such images already, but not all.
2. Image Reconstruction Error: GSPLAT-based reconstructions can be noisy where regions are poorly represented by the learned model. Even areas, however, that appear visually consistent to the human eye are not quite the same as the captured image–leading to significant differences when processed by object detection.
3. Uninteresting Environmental Change: Lighting conditions are a good example of uninteresting change that can impact object detection. Furniture being moved, even slightly through regular use, is another. These will generate small numbers of false positive pixels where object detection estimates unexpectedly differ.

The first evaluation, therefore, focuses on understanding the data impact. What are the reductions in image, pixel, and object bounding box counts due to the change detection filter?

#### 4.3.1 Point Counts

**FIGURE 12:**
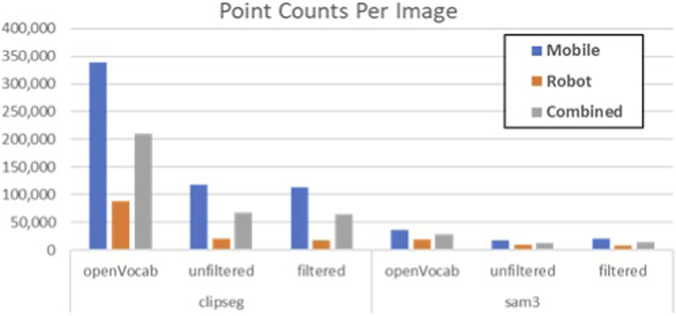
Average number of pixels that exceed threshold per image per algorithm.

Figure 12 plots the point counts per image for each algorithm. This is the number of pixels that exceed the 0.1 threshold, normalized by the number of images evaluated. Table 1 highlights the percentage change in the number of pixels between the delta change image and the baseline segmentation algorithm without change.

**TABLE 1:** Percentage of change pixels per image relative to the number of open vocab pixels exceeding threshold.

	clipseg		sam3	
Algorithm	Change	Change/ Blur	Change	Change/ Blur
Mobile	35%	33%	47%	56%
Robot	24%	20%	46%	42%
Combined	32%	31%	47%	51%

As expected, change detection significantly reduces the number of pixels–although the amount also varies significantly by algorithm. Compared to the open vocabulary-based methods, change detection reduces CLIPSEG pixel counts by 68%, but only 53% with SAM3. Removing the blurriest 30% of images has a negligible impact on pixel counts per image for CLIPSEG (1%), but actually increases the pixel count per image with SAM v3. This makes sense because the images removed are largely uninteresting and lack significant objects of interest. So, although the total pixel counts drop ∼10% by removing 30% of the images, the per image count can actually go up.

#### 4.3.2 Nonzero Image Count

Most images do not contain significant changeWithout sources of error, the numbers of images crossing the 0.1 threshold should decrease significantly when change detection is applied. Unfortunately, this is not what we are observing, particularly with SAM v3 (Table 2).

**TABLE 2:** Number of images where the delta change image was non-zero.

	CLIP-Seg			SAM v3		
Algorithm	OpenVocab	Unfiltered	Filtered	OpenVocab	Unfiltered	Filtered
General clutter	2185	1657	1174	484	441	322
Small items	1798	1272	919	1550	1522	1074
Total	6782	5162	3563	3132	3035	2123

**FIGURE 13:**
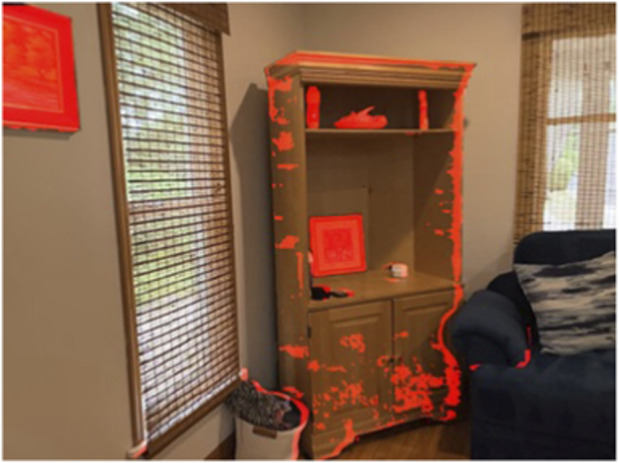
The delta change image for SAM v3 often contains pixels along the edges of objects where there were differences between segmentation on the real vs. rendered images.

Although CLIP-Seg (Change) does drop ∼26% of images compared to CLIP-Seg (OpenVocab), SAM3 (Change) only drops 3% of images. This is a case where SAM’s aggressive expansion to object edges is actually counter-productive as demonstrated in Figure 13. In this case, there is likely a small localization error and as a result, pixels on object boundaries are less likely to be removed–leading to more images with noisy pixels crossing the threshold.

#### 4.3.3 Box counts

With the number of pixels being reduced, we expected to also see a similar reduction in the number of detected object bounding boxes. Perhaps surprisingly, the impact is greatest on SAM v3. Whereas the total number of boxes per image drops by 42% when using CLIPSEG, the drop is 78% with SAM v3 (Table 3).

**TABLE 3:** Box counts per image for each prompt.

Prompt	Clipseg			sam3		
	openVocab	Change	Change/blur	openVocab	Change	Change/blur
*dishes*	0.23	0.18	0.16	0.26	0.05	0.07
*clothing*	0.45	0.45	0.45	0.37	0.09	0.10
*electronics*	1.27	0.89	0.77	8.41	1.93	1.71
*trash*	1.86	0.84	0.81	2.24	0.75	0.64
*general clutter*	1.61	0.86	0.86	0.96	0.31	0.34
*small items*	0.96	0.54	0.55	8.67	1.72	1.73
Total	1.12	0.65	0.63	3.84	0.87	0.84

The reason for the larger drop with SAM is the profligacy of the 0.1 threshold on some queries. Note that “small items” and “electronics” generate more than 8 boxes per image on average in the tested environments. This is significantly higher than the almost 2 boxes per image compared to “general clutter” and “trash”, the two prompts with the highest numbers of boxes when using CLIPSEG.

### 4.4 Evaluation of clustering

**FIGURE 14:**
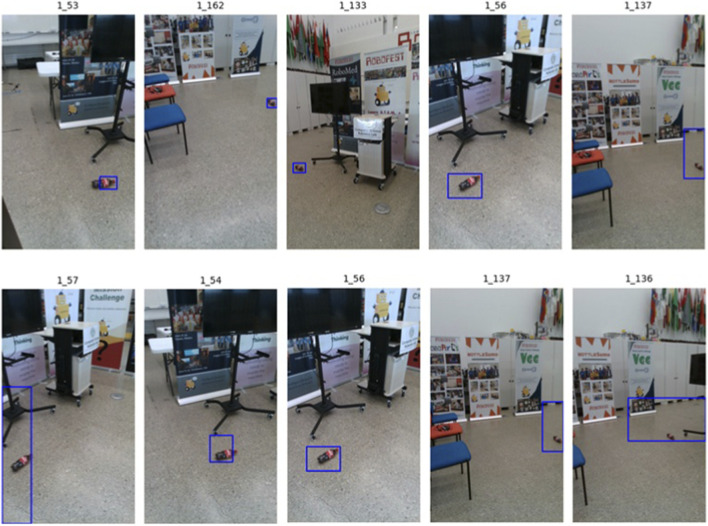
Example bounding boxes drawn around a detected cluster in the environment. Only those boxes with an IOU>0.25 are counted when determining if the cluster was valid.

Clusters are grasp targets for the service robot. They indicate objects that should be picked up and put away. Whereas the previous section focused entirely on reducing the number of candidate objects, this section uses hand labeling to evaluate performance. For each cluster, all images containing pixels included with in the cluster are identified and a bounding box around the cluster is drawn on the image (Figure 14). These images are then manually reviewed. If >50% of the images have bounding boxes with IOU>0.25 around an object that has been moved since the baseline run, the object is marked as a positive example. All other clusters are marked as negative. This IOU was selected because it is enough to get a robot within view of the target, at which point SAM or CLIPSEG must be re-applied to guide grasping.

Using the labeled clusters, we evaluate the impacts of different queries, different filters, and the accuracy of object detection.

#### 4.4.1 Impact of different queries and data collection devices

Table 4 demonstrates that even with the significantly reduced numbers of pixels and bounding boxes being used with the change image, the number of changed objects still increases with both the CLIP-Seg and SAM-v3 algorithms. Note that not all classifiers are capable of detecting all objects. It is also difficult to estimate in advance exactly which objects a generic prompt should detect (i.e., should a blanket or backpack qualify as a “small item”?). As such, we have focused on object counts rather than a more traditional recall score that requires knowing the total number of positive examples in the environment.

**TABLE 4:** Counting the number of correctly detected objects across all environments by different prompts and algorithms.

	CLIP-Seg			SAM v3		
Prompt	OpenVocab	Change	% increase	OpenVocab	Change	% increase
*clothing*	2	2	0.0%	2	1	−50.00%
*dishes*	3	3	0.0%	1	1	0.00%
*electronics*	8	9	12.5%	16	20	25.00%
*general clutter*	16	35	118.8%	0	0	0.00%
*small items*	31	36	47.2%	53	60	13.21%
*trash*	22	19	−13.6%	21	17	−19.05%
**Combined**	82	104	39.1%	93	99	6.45%

This is also corresponding jump in precision by applying change detection (Table 5).

**TABLE 5:** The calculated precision per query for all extracted clusters.

	CLIP-Seg			SAM v3		
Prompt	OpenVocab	Change	Delta	OpenVocab	Change	Delta
*clothing*	4.3%	4.9%	0.6%	28.6%	25.0%	−3.6%
*dishes*	15.8%	21.1%	5.3%	25.0%	66.7%	41.7%
*electronics*	8.5%	14.1%	5.6%	18.0%	20.2%	2.2%
*general clutter*	6.7%	26.4%	19.7%	0.0%	0.0%	0.0%
*small items*	18.7%	51.2%	32.5%	27.7%	51.5%	23.8%
*trash*	19.0%	33.3%	14.3%	81.5%	100.0%	18.5%

These results also highlight how picking the right query (or basket of queries) still requires trial and error. Two of the queries, ‘clothing’ and ‘dishes’, should have been ideal for use in the home environment. There were examples of dishes in 6 trials, sometimes more than one per trial, and a similar number for clothing, but SAM v3 only correctly identified 2 clothing items and 1 example of dishes even with a 0.1 threshold. By contrast, ‘small items’ picked up most of the dishes and the clothing examples, with both CLIP-Seg and SAM recognizing most examples across all trials.

CLIP-Seg and SAM-v3 also demonstrate significant differences between queries. The ‘general clutter’ prompt was initially selected because of its utility with CLIP-Seg, but SAM-v3 identified 0 objects using that prompt. ‘Electronics’ also showed lopsided performance, but this time with SAM-v3 detecting many more examples.

#### 4.4.2 Applying filtering to improve precision

The previous section was unfiltered. As discussed in Section 3.2.3, however, clusters can be scored by a number of different methods: calculating the mean detection score of each cluster (prob-mean), calculating the percentage of images with recognized boxes divided by the total number of images that can see that object (Pct-Valid), and using an LLM to identify whether or not the object should be picked up (LLM-Pickup). There is also a combined filter multiplying the Pct-Valid and LLM-Pickup results. These methods each generate a score between [0,1]. By varying the threshold of acceptance, the precision can be increased while potentially reducing the number of objects accepted.

Focusing on just the “small items” query, Table 6 explores the impact of different filters on achievable precision. The first two columns (Max Count), identify the precision per filter associated with the highest object count achievable by all filters. For instance, as reported earlier, CLIP-Seg (Change) can detect 31/36 objects with without filtering in OpenVocab/Change conditions, but the LLM-Pickup scores some objects with a zero, so the reported precisions for all filters are for 29 (OpenVocab) and 35 objects (Change) to enable effective comparison. SAM-v3 is reduced by 1 in both conditions.

**TABLE 6:** Recorded precision on labeled data after applying different types of filters.

Filter	OpenVocab - max count	Change - max count	Change - match OV
CLIPSeg			
*Unfiltered*	18.7%	51.2%	51.2%
*Prob-Mean*	18.8%	61.2%	76.6%
*Pct Valid*	18.5%	62.9%	72.7%
*LLM-Pickup*	37.9%	86.9%	94.2%
*Combo*	55.6%	88.9%	94.1%
SAM v3			
*Unfiltered*	27.7%	51.5%	51.5%
*Prob-Mean*	31.3%	54.1%	56.4%
*Pct Valid*	39.2%	58.2%	68.3%
*LLM-Pickup*	57.7%	65.7%	73.5%
*Combo*	66.7%	75.9%	81.4%

Both the Prob-Mean and Pct-Valid filters raise performance over baseline in all conditions–but the range of improvement is generally small. The low impact of the Prob-Mean filter in particular is likely due to our using the object detection algorithms for purposes other than typically intended. High object scores are not correlated with “changed” objects, rather with word associations. The Pct-Valid filter does slightly better with the SAM-v3 algorithm, but still by <10%.

The LLM-Pickup filter is what delivers the greatest value, boosting the precision of both the OpenVocab and Change conditions. The SAM-v3 (OpenVocab) precision is boosted by 30%, while the CLIP-Seg (Change) precision is boosted by 24%. Furthermore, combining the LLM-Pickup results with the Pct-Valid filter continues to boost precision, raising all 4 filters to their highest values while maximizing the object count.

The last column in the table (Match OV) highlights what would happen to precision if we selecting the threshold to match the object count in both the Change and OpenVocab conditions. With this change, the Pct-Valid filter increases the precision by 10% with both CLIP-Seg and SAM-v3. The LLM-Pickup filter pushes the precision of CLIP-Seg (Change) to 94% and SAM-v3 to 81%. Note that the SAM-v3 filter, however, has significant room for improvement with a higher starting object count. By fixing the object count for SAM-v3 to 29 (same as CLIP-Seg, OpenVocab), the precision also improves to >90%.

In general, this demonstrates the potential power of LLMs. The ability to inject context into robot decision making, in this case recognizing if objects should be picked up or not, can make a significant difference in reaching minimum viable precision levels.

#### 4.4.3 Impact of blur filters

The blur filter was introduced earlier after observing that a significant number of rendered images were blurry–particularly in the mobile phone data. Although early indicators of pixel counts and boxes looked appropriate, Table 7 highlights the mixed impact of blur filters in evaluations with labeled data. These evaluations were conducted only on the mobile phone collected data where localization seemed to struggle more frequently–resulting in higher offset and blur.

**TABLE 7:** The impact of blur filters on object counts and precision.

	CLIP-Seg		SAM-v3	
	Object count	Precision	Object count	Precision
*OpenVocab*	15	0.169014	13	0.204545
*Change*	19	0.227778	20	0.442857
***Blur***	18	0.282443	12	0.418182

Object counts using both CLIP-Seg and SAM-v3 are reduced, with the latter dropping by more than 1/3. Precision actually rises 5.5% with CLIP-Seg but drops 3% with SAM-v3. This suggests that blur-filtering might be situationally useful, but is not a guaranteed boost to overall performance.

#### 4.4.4 Object localization precision

In addition to recognizing that a cluster has changed, it is also important to evaluate the precision with which objects are successfully localized so that a robot can find them and interact with them as required for the task. To this end, the testing in the office environment also included measuring the pose of the object centroid in 3D space relative to a known origin. Robot generated estimates of pose were then compared to the hand measurements.

On average, robot estimated poses of objects were 0.26-m from the measured centroid. One object exceeded 0.6-m, and many objects were estimated as being below the ground plane by 10–20 cm.

These results are reasonable. As will be discussed further in Section 5.1, a robot should deploy a real-time model of open-vocabulary object detection to re-identify the object before attempting to grasp it. The only requirement is that the pose not be too far away for it to be visible in the image. Re-identification will account for cluster estimation errors as well as objects that might be bumped by people or the robot itself.

## 5 Discussion

The primary objective of this research is to realize a domestic service robot capable of autonomous environment maintenance—specifically identifying and retrieving misplaced objects. To achieve this, we leverage foundation models, such as open-vocabulary object detection and segmentation, to bypass the traditional bottleneck of environment-specific object categorization. By employing generic semantic prompts like “small items” or “trash,” our framework enables a robot to interact with an unpredictable array of household objects without requiring manually labeled training data. While traditional classifiers like YOLO remain effective for static, predefined object sets, this work demonstrates that for robots operating in the unstructured and evolving environments of human homes, change detection integrated with foundation models offers a superior path toward improved precision and scalable deployment.

Experimental results across both mobile phone and robotic data collection platforms validate this approach. The integration of change detection reduced pixel-level noise by over 70% using CLIP-Seg and 50% with SAM-v3. Bounding box redundancies were similarly mitigated, falling by 70% and 40%, respectively. Crucially, these reductions in data complexity occurred while simultaneously increasing the total number of detected objects and improving overall precision. In the case of CLIP-Seg, the total number of detected objects increased by 27% across all queries, with precision gains of up to 30% for specific tasks.

However, visual change detection alone often lacks the semantic precision required for reliable robotic intervention. To address this, we introduced Large Language Models (LLMs) as a secondary foundational layer to inject task-specific context. By utilizing an LLM (Llama-scout) to evaluate the feasibility of picking up a detected object, we significantly boosted system reliability. This contextual filtering allowed change-detection-equipped CLIP-Seg to achieve 94% precision, while SAM-v3 reached 81% precision with a substantially higher object count. These improvements directly enable practical applications in service robotics, most notably in autonomous cleaning and remote patient monitoring (RPM).

In summary, these detection improvements enable new capabilities in service robotics. The following sections discuss two applications of the proposed change detection system that we are now beginning to investigate: Cleaning with a Mobile Manipulator, and Monitoring Change for Health Tracking.

### 5.1 Cleaning with a mobile manipulator

**FIGURE 15:**
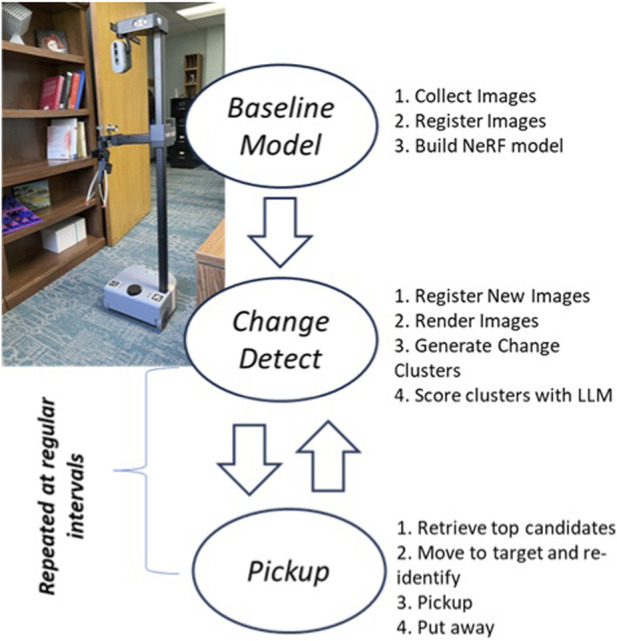
The proposed robotic cleaning service has 3 stages: baseline model creation, change detection, and grasping. Two out of three are fully completed.

Using change detection to pick up objects in a house is a 3-stage process: building a baseline model of the home, then repeatedly identifying objects for cleaning, and picking them up (Figure 15). For now, we are assuming the robot is putting the objects into a box for people to take care of later. However, we are actively investigating approaches for recognizing where objects belong from a recorded baseline.

- Baseline Construction–constructed only at a user command, this process would be run infrequently to make sure the image reconstruction model is good enough. This process has already been automated as part of this current work. A ROS2 node handles the waypoint navigation through the environment, storing images to disk. Then a separate process invokes the necessary COLMAP and NeRFStudio commands to create the baseline model and file structure.
- Change Object Identification–the process of extracting clusters and applying LLM-based filtering to set pick-up goals for the mobile robot. This process would be repeated as needed. At present, the process is started manually, but the actual navigation, data collection, change cluster extraction and scoring work has already been fully automated. One remaining challenge with this operation is the speed of image registration–which grows with the number and complexity of images in both the baseline and new datasets. Breaking the problem into smaller chunks (i.e., room by room) with hundreds instead of thousands of images will reduce registration costs significantly for whole house operations.
- Picking Up Objects–the final step of picking up the object has a number of remaining technical challenges. Current work has a ROS2 Change Server node that indicates targets. The robot (Stretch-2) then navigates to the region of interest using Nav2, re-identifies the grasp target with DINO, and then calculates the grasp location using the head-mounted Intel RealSense D435i depth camera. This approach is suitable for navigating to objects in open floor spaces, but is not great at retrieving objects near walls, or under (or on top of) furniture. It also struggles with rigid objects that need to be grasped from a particular angle (e.g., bottle). Future work will seek to improve these capabilities, possibly incorporating LLM-based planning as a solution.

In general, the proposed cleaning solution shares some similarities with a robot vacuum. The robot vacuum does not need a powerful motor to pick up all the dust in a single round because it runs much more frequently than a person doing the same task. If a robot can only find one object that has changed this round, then it still succeeds in making the area cleaner. Then when it tries again later, it will likely find some of the objects it has missed. Lighting conditions, registration errors, robot motion differences, etc., are all potential sources of error between runs that could change the scores of the object detection system and allow the robot to find targets missed on the previous pass.

### 5.2 Change detection to support remote patient monitoring

In healthcare, detecting change is generally about monitoring a patient’s condition (Agency for Healthcare Research and Quality, 2014). This means establishing a qualitative baseline of mental and physical health, and then recording regular observations of eating habits, sleep patterns, mobility, communications, social interactions, etc. The challenge is to detect the early signs or negative trends before serious health events occur and hospitalization is required. Sensing can absolutely help, but has generally focused on detecting human activity. For example, Wu et al. (Wu, et al., 2021) highlight a network of motion, depth, and bed sensors spread throughout a residence to monitor behavioral patterns. Others have used cameras for similar types of activity monitoring (Aramendi, et al., 2018).

These efforts highlight the need for RPM solutions and the existing focus on human activity. But what about other indicators of cognitive or physical wellbeing? Can we ultimately estimate anything about the current state of an individual through repeated observations of their environment? In interviews Martinson et al. conducted with resident nurses in 2016 (Martinson, et al., 2016), one of the recorded observations was that “elderly people with limited mobility, do a really good job of building a nest. So, they have their chair, and they have the coffee table [or] the TV tray right here. And you can really tell, based on where things are placed what their limitations are.” Outside of range of motion, depression has been previously connected with “household chaos” or messy rooms (Marsh, et al., 2020; Pattemore and Pineda, 2022). Alzheimers and dementia are also correlated with increased disorder, and specific problems like objects being left in unusual places or lost entirely (McGarrigle, et al., 2019; Alzheimers Association, 2017).

These studies suggest that tracking change to objects and/or furniture in a patient’s living space could be used to infer important aspects of their physical or mental conditions. Whereas the work discussed so far has focused on being able to pick-up objects, the implications for eldercare is also tremendous. Could a vacuum cleaner equipped with change detection recognize what objects have been used over time? Or maybe the cleaning robot from Section 5.1 provides a supplemental service for at-risk patients–reviewable only be a doctor. Even a nurse holding a mobile phone could collect the necessary information. It is an exciting new opportunity within the field of RPM as change detection could offer significant potential for early detection and treatment options without requiring direct observations of the patient–thereby reducing perceived invasiveness of the monitoring.

### 5.3 Summary

In the long term, we expect that change detection is a decisive enabler for home robots–whether these robots are cleaning the house or providing remote patient monitoring services. Foundation models like Open-Vocabulary Object Detection and Large Language Models are making it possible to develop practical solutions that have long evaded robotics. Now without building large labeled data sets or limiting robots to very small groups of items, change detection enables interaction with many common objects even without knowing exactly what they are. Furthermore, we have demonstrated that these approaches work without expensive sensors. Maybe to save development costs, a designer would have a person scan their own home in the future using their phone, sending the results to the robot for clean up? It opens up intriguing new possibilities for human-robot collaboration as well.

And just like a human, the robot only needs to recognize that something has changed.

## Data Availability

The raw data supporting the conclusions of this article will be made available by the authors upon request.
